# Impact of the baseline insulin resistance surrogates and their longitudinal trajectories on cardiovascular disease (coronary heart disease and stroke): a prospective cohort study in rural China

**DOI:** 10.3389/fendo.2023.1259062

**Published:** 2023-12-22

**Authors:** Shulin Wang, Xianghui Zhang, Mulatibieke Keerman, Heng Guo, Jia He, Remina Maimaitijiang, Xinping Wang, Rulin Ma, Shuxia Guo

**Affiliations:** ^1^ Department of Public Health, Shihezi University School of Medicine, Shihezi, China; ^2^ Department of National Health Commission Key Laboratory of Prevention and Treatment of Central Asia High Incidence Diseases, The First Affiliated Hospital of Shihezi University School of Medicine, Shihezi, China

**Keywords:** cardiovascular disease, cohort study, metabolic score for insulin resistance, triglyceride-glucose index, trajectory analysis

## Abstract

**Background:**

This study aimed to assess the association of baseline insulin resistance (IR) surrogates and their longitudinal trajectories with cardiovascular diseases (CVD) to provide a useful reference for preventing CVD.

**Methods:**

This study was a prospective cohort study conducted in the 51st Regiment of the Third Division of Xinjiang Corps. A total of 6362 participants were recruited in 2016 to conduct the baseline survey, and the follow-up surveys in 2019, 2020, 2021, and 2022. The Kaplan–Meier method was used to estimate the cumulative incidence of CVD according to the baseline IR surrogates of metabolic insulin resistance score (METS-IR) and triglyceride-glucose (TyG) index. Cox regression models were used to assess the association between the baseline IR surrogates and CVD. The impact of the longitudinal trajectories of the IR surrogates on CVD was analyzed after excluding those with IR surrogate data measured ≤2 times. Based on the group-based trajectory model (GBTM), the trajectory patterns of IR surrogates were determined. The Kaplan-Meier method was used to estimate the cumulative incidence of CVD in each trajectory group of METS-IR and TyG index. Cox regression models were used to analyze the association between different trajectory groups of each index and CVD. In addition, the Framingham model was utilized to evaluate whether the addition of the baseline IR surrogates increased the predictive potential of the model.

**Results:**

Baseline data analysis included 4712 participants. During a median follow-up of 5.66 years, 572 CVD events were recorded (mean age, 39.42 ± 13.67 years; males, 42.9%). The cumulative CVD incidence increased with the ascending baseline METS-IR and TyG index quartiles (Q1–Q4). The hazard ratio and 95% confidence interval for CVD risk in Q4 of the METS-IR and TyG index were 1.79 (1.25, 2.58) and 1.66 (1.28, 2.17), respectively, when compared with Q1. 4343 participants were included in the trajectory analysis, based on the longitudinal change patterns of the METS-IR and TyG index, the following three trajectory groups were identified: low-increasing, moderate-stable, and elevated-increasing groups. Multivariate Cox regression revealed that the hazard ratio (95% confidence interval) for CVD risk in the elevated-increasing trajectory group of the METS-IR and TyG index was 2.13 (1.48, 3.06) and 2.63 (1.68, 4.13), respectively, when compared with the low-rising group. The C-index, integrated discrimination improvement value, and net reclassification improvement value were enhanced after adding the baseline METS-IR and TyG index values to the Framingham model (*P*<0.05).

**Conclusions:**

Elevated baseline IR surrogates and their higher long-term trajectories were strongly associated with a high risk of CVD incidence in Xinjiang’s rural areas. Regular METS-IR and TyG index monitoring can aid in the early detection of CVD-risk groups.

## 1 Introduction

Cardiovascular diseases (CVD) are associated with high morbidity and mortality rates. Approximately one-third of deaths worldwide are attributable to CVD, which ranks first among chronic non-communicable diseases in terms of fatalities (1). With approximately 330 million people afflicted, the incidence of CVD is rising in China, as is the illness burden associated with CVD (2). Therefore, early detection of persons at risk for CVD through effective screening measures and the development of preventative and treatment strategies are critical.

Insulin resistance (IR) represents one of the key pathogenic mechanisms of CVD, which promotes CVD development through numerous physiological and biochemical pathways that accelerate and exacerbate the CVD processes (3). The homeostasis model assessment of insulin resistance (HOMA-IR) is a common approach for assessing IR; however, its practical application is restricted because insulin is not a conventional measure, and the test is expensive (4). The recently introduced metabolic score for insulin resistance (METS-IR) and triglyceride-glucose (TyG) index has the advantages of being less expensive and simpler than HOMA-IR and has been proven to be useful in assessing IR (5, 6). The correlations of METS-IR and TyG index with CVD risk have been validated in various cohorts (7–9). However, most of the previous studies analyzed the association of METS-IR and TyG index with CVD using single measurement data, neglecting the influence of their dynamic changes on CVD during the follow-up period.

Although the prevalence of CVD is substantial in the rural areas of Xinjiang, investigations involving IR surrogates in this population are limited (10). To provide a reference and theoretical foundation for CVD risk prediction and targeted prevention and control, this study used a prospective cohort to assess the relationship between the baseline and trajectories of IR surrogates with CVD.

## 2 Materials and methods

### 2.1 Study population

**Figure 1:**
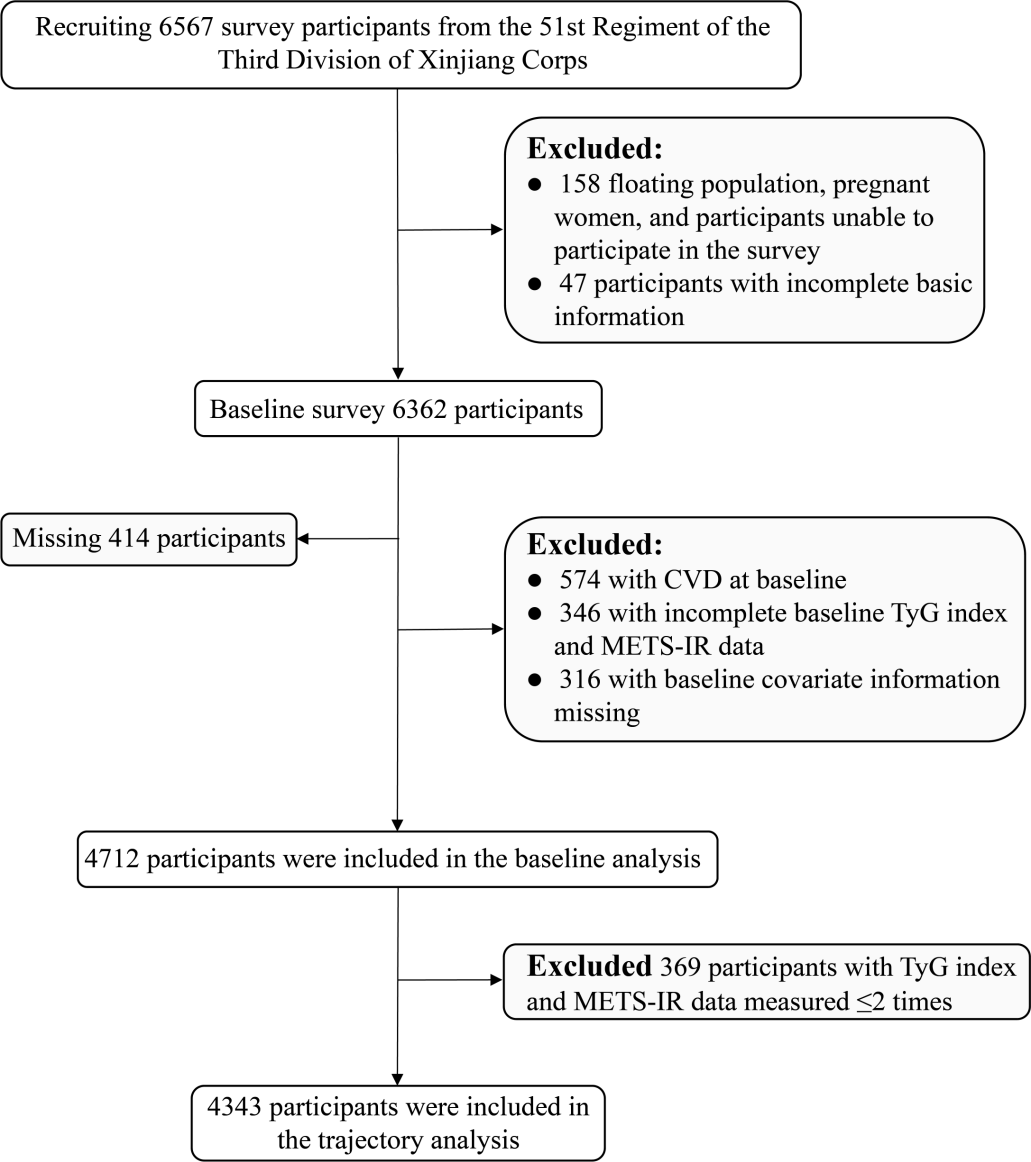
Flow chart of the inclusion and exclusion criteria of the rural Xinjiang population. (*CVD*, cardiovascular diseases; *TyG* index, triglyceride-glucose index; *METS-IR*, metabolic insulin resistance score).

The 51st Regiment of the Third Division of Xinjiang Corps was chosen as the survey location using a typical sampling method. In total, 6567 adult residents of the 51st Regiment’s five squadrons for over a year were chosen as survey participants using a stratified cluster random sampling method. The baseline survey was conducted in 2016, and the follow-up surveys in 2019, 2020, 2021, and 2022. The contents of the follow-up survey were consistent with those of the baseline survey. At baseline, 6362 participants were surveyed after excluding the floating population, pregnant women, those unable to participate in the survey (n=158), and those with incomplete basic information (n=47). During the follow-up period, 414 participants were lost to follow-up (follow-up rate: 93.5%). After excluding baseline CVD participants (n=574), baseline METS-IR and TyG data incomplete participants (n=346), and baseline covariate information missing participants (n=316), 4712 participants were included in the study to look into the association between the baseline IR surrogates and CVD. To examine the impact of the longitudinal trajectories of the IR surrogates on CVD, 4343 participants were included after excluding those with METS-IR and TyG index data measured ≤2 times (n=369) (**Figure 1**).

### 2.2 Data collection

#### 2.2.1 General information collection

Professionally trained personnel administered the questionnaire and conducted physical examinations for the participants. The face-to-face survey using a questionnaire was conducted to collect demographic information, lifestyle habits (including exercise frequency, smoking habits, and drinking habits), and family and personal medical histories. Physical examination was conducted using standard methods to measure height, weight, waist circumference (WC), and other health indicators. Height and weight measuring instrument was used to measure height and weight and the body mass index (BMI) was calculated. The participants in the measurement stood straight, leaned on the column, removed their shoes and bulky apparel, and fixed their gaze ahead while keeping both eyes flat. The WC was measured by a flexible ruler at the horizontal position of the axillary line between the lower edge of the costal arch and the midpoint of the iliac crest. The blood pressure was measured twice in the seated position at an interval of 5 mins using an electronic sphygmomanometer (OMRON HEM-7051, Omron, Dalian Co., Ltd.), and the average result was recorded.

#### 2.2.2 Laboratory examination

Venous blood samples (5 mL) were collected from the anterior aspect of the participants’ elbows while they were fasting on the day of the physical examination. Biochemical indicators such as fasting plasma glucose (FPG), triglycerides (TG), total cholesterol (TC), low-density lipoprotein cholesterol (LDL-C), and high-density lipoprotein cholesterol (HDL-C) were measured using the OLYMPUS 2007 automatic biochemical analyzer. The METS-IR was calculated as “Ln [(2×FPG (mg/dL))+TG (mg/dL)]×BMI/Ln [HDL-C (mg/dL)]” (6); the TyG index was calculated as “Ln [TG (mg/dL)×FPG (mg/dL)/2]” (5).

### 2.3 Relevant definitions

(1) Hypertension: systolic blood pressure ≥140 mmHg and/or diastolic blood pressure ≥90 mmHg, self-reported hypertension, or self-reported use of antihypertensive medications within 2 weeks (11);
(2) Prediabetes and diabetes: prediabetes was defined as 5.6 mmol/L≤FPG≤ 7.0 mmol/L; diabetes was defined as FPG ≥7.0 mmol/L or history of diabetes (12);
(3) Smoking: continuous or cumulative smoking for ≥6 months (13);
(4) Drinking: ≥2 times/month for >6 months (14);
(5) Exercise frequency: regular exercise (≥3 times/week, exercise time: ≥30 min/session); occasional exercise (<3 times/week, exercise time: <30 min/session); and almost no exercise (<1 time/week) (15).

### 2.4 Diagnostic criteria for cardiovascular disease

The study outcome events were new-onset coronary heart disease (International Classification of Diseases, Tenth Revision [ICD-10]: I20–I25) or stroke (ICD-10: I60–I64 and I69) during the follow-up period (16). Data on the outcome events were obtained from questionnaires, public primary hospital case files, and social security information. The time of the first outcome event was chosen as the end event if several outcome events occurred in the same participant. Those who self-reported the outcome events had to present documentation of the clinical diagnosis made at a county-level hospital or higher.

### 2.5 Statistical analysis

#### 2.5.1 Descriptive analysis

Continuous variables are presented as means ± standard deviations, and the independent samples t-test was used for inter-group comparisons. Categorical variables are presented as frequencies and composition ratios, and the χ^2^-test was used for inter-group comparisons.

#### 2.5.2 Analysis of the association between baseline IR surrogates and CVD

The Kaplan–Meier method was used to estimate the cumulative incidence of CVD in the quartile groups of the baseline METS-IR and TyG index, and the log-rank test was performed to determine any difference in the cumulative incidence of CVD in the quartile groups. Cox regression models were used to analyze the association between the quartile groups of each index and CVD onset. The covariates of the multivariate Cox regression model included age, sex, education level, exercise frequency, WC, smoking, drinking, family history of CVD, hypertension, HDL-C, and LDL-C. Subgroup analyses were for the following variables: sex, age (<45 and ≥45 years), BMI (<28 and ≥28 kg/m^2^), and hypertension (yes and no). The interaction between the subgroups was evaluated using the likelihood ratio test. Sensitivity analysis was performed to verify the consistency of the results. Initially, to reduce the possibility of reverse causality, the participants who experienced an outcome event within the first year of the follow-up were excluded. Secondly, the Cox regression analysis was repeated after excluding the population with hypertension, considering that this population might affect the findings. Third, sensitivity analysis was performed after excluding the population with dyslipidemia. Finally, a sensitivity analysis was conducted, excluding individuals with prediabetes and diabetes. The Framingham CVD risk score model (17) was used to evaluate the incremental predictive value of the baseline METS-IR and TyG index and to assess whether their addition could improve the predictive power of the model based on the C-index, net reclassification improvement (NRI), and integrated discrimination improvement (IDI).

#### 2.5.3 Analysis of the association between IR surrogates’ trajectories and CVD

The group-based trajectory model (GBTM) (18) identifies population subgroups with similar development trends over time in the cohort based on multiple repeated measurement data and depicts the longitudinal development trajectory curve of each subgroup. In this study, the trajectory patterns of the METS-IR and TyG index of the participants were determined using the above model. We repeatedly tested models with groups ranging from 2 to 5 until the ideal group of trajectories was identified. Following this, the variation order of each trajectory (linear, quadratic, and cubic) was modified for repeated testing so that the estimated values of each parameter reached the significance level; this allowed for the determination of the curve shape of each trajectory group in the final model. The following criteria were used to determine which trajectory-fitting model was the best: (1) absolute Bayesian information criterion minimization; (2) average posterior probabilities of each trajectory subgroup >0.70; and (3) followed the explanation of the expert theory. The METS-IR and TyG index trajectories were subsequently divided into the following three groups based on the results of numerous iterations of the fit: low-rising group, moderate-stable group, and elevated-increasing group. This has been presented in the additional table files (**Supplementary Table 1**). To assess the effect of the METS-IR and TyG index trajectory groups on CVD, the Kaplan–Meier method was used to calculate the cumulative incidence of CVD in each trajectory group. The log-rank test was used for comparison between the groups, and the Cox regression model was used to examine the impact of the different trajectory groups of the METS-IR and TyG index on CVD. The sensitivity analysis method for the different trajectory groups of the METS-IR and TyG index was the same as that used for the baseline data analysis.

All data were analyzed using SPSS 26.0, Stata 17.0, and R 4.1.2; *P*-values <0.05 were considered statistically significant for all analyses.

## 3 Results

### 3.1 Baseline characteristics

Among the 4712 participants in this study, 42.9% were male, and the average age was 39.42 ± 13.02 years. The FPG, TG, TC, HDL-C, LDL-C, BMI, and WC differed significantly between the CVD and non-CVD groups (*P*<0.05). Smoking habits, family history of CVD, and hypertension were reported in 17.1%, 19.4%, and 40.9% of the participants in the CVD group, respectively, which were significantly higher than those reported in the non-CVD group participants (*P*<0.05). The percentage of the CVD group participants with no formal education and almost no exercise was higher than that of the whole population (**Table 1**).

**Table 1:** Baseline characteristics of Xinjiang’s rural population according to the incidence of cardiovascular diseases.

Variables	CVD group (n=572)	Non-CVD group (n=4140)	Total (n=4712)	t/χ^2^ value	*P* value
Male (%)	205 (35.8)	1816 (43.9)	2021 (42.9)	13.215	<0.001
Age (years)	49.79 ± 12.93	37.98 ± 12.36	39.42 ± 13.02	20.581	<0.001
Education level				33.749	<0.001
Illiterate/semi-illiterate (%)	284 (49.7)	1564 (37.8)	1848 (39.2)		
Junior high school and below (%)	242 (42.3)	2278 (55.0)	2520 (53.5)		
High School and above (%)	46 (8.0)	298 (7.2)	344 (7.3)		
Exercise frequency				16.816	<0.001
Regular exercise (%)	171 (29.9)	1449 (35.0)	1620 (34.3)		
Occasional exercise (%)	28 (4.9)	338 (8.2)	366 (7.8)		
Almost no exercise (%)	373 (65.2)	2353 (56.8)	2726 (57.9)		
Systolic blood pressure (mmHg)	137.86 ± 24.59	126.37 ± 19.16	127.77 ± 20.24	10.736	<0.001
Diastolic blood pressure(mmHg)	80.96 ± 15.55	74.98 ± 11.82	75.71 ± 12.49	8.844	<0.001
FPG (mmol/L)	4.97 ± 1.27	4.68 ± 0.98	4.72 ± 1.03	5.105	<0.001
TG (mmol/L)	1.59 ± 0.63	1.38 ± 0.64	1.41 ± 0.64	7.207	<0.001
TC (mmol/L)	4.76 ± 1.06	4.62 ± 1.04	4.64 ± 1.04	3.044	0.002
HDL-C (mmol/L)	1.34 ± 0.36	1.42 ± 0.38	1.41 ± 0.38	-5.078	<0.001
LDL-C (mmol/L)	2.74 ± 0.89	2.57 ± 0.89	2.59 ± 0.90	4.250	<0.001
BMI (kg/m^2^)	28.30 ± 4.14	26.46 ± 4.18	26.69 ± 4.22	9.842	<0.001
WC (cm)	95.57 ± 10.33	90.67 ± 11.30	91.27 ± 11.30	10.499	<0.001
TyG index	8.65 ± 0.46	8.43 ± 0.53	8.46 ± 0.52	10.379	<0.001
METS-IR	41.67 ± 7.14	37.83 ± 7.15	38.30 ± 7.26	12.021	<0.001
Smoking (%)	98 (17.1)	571 (13.8)	669 (14.2)	4.604	0.032
Drinking (%)	27 (4.7)	156 (3.8)	183 (3.9)	1.221	0.269
Hypertension (%)	234 (40.9)	869 (21.0)	1103 (23.4)	111.215	<0.001
Family history of CVD (%)	111 (19.4)	478 (11.5)	589 (12.5)	28.385	<0.001

(CVD, cardiovascular diseases; FPG, fasting plasma glucose; TG, triglycerides; TC, total cholesterol; LDL-C, low-density lipoprotein cholesterol; HDL-C, high-density lipoprotein cholesterol; BMI, body mass index, WC, waist circumference; METS-IR, metabolic insulin resistance score; TyG index, triglyceride-glucose index).

### 3.2 Association between baseline IR surrogates and CVD

**Figure 2:**
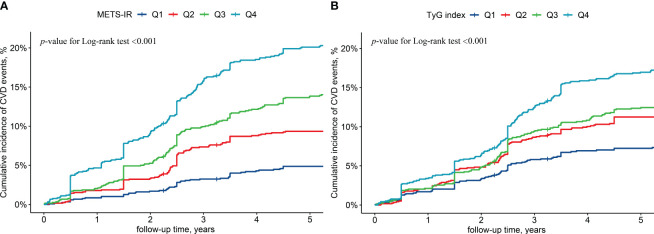
Cumulative cardiovascular diseases incidence by baseline metabolic insulin resistance score **(A)** and triglyceride-glucose index **(B)**. (*METS-IR*, metabolic insulin resistance score; *TyG* index, triglyceride-glucose index; *CVD*, cardiovascular disease; *Q1*, Quartile 1; *Q2*, Quartile 2; *Q3*, Quartile 3; *Q4*, Quartile 4).

The cohort had a cumulative CVD incidence of 12.1% during a median follow-up period of 5.66 years. In total, 572 participants had their first CVD event during 23,614.71 person-years of follow-up (24.22/1000 person-years). With the ascending baseline METS-IR and TyG index quartiles, the cumulative incidence of CVD increased considerably and peaked in the Q4 group (**Figure 2**). After controlling for the baseline age, sex, education level, exercise frequency, WC, smoking, drinking, family history of CVD, hypertension, HDL-C, and LDL-C, the METS-IR and TyG index were found to be associated with an increased risk of CVD. Compared to Q1, the hazard ratio (HR) (95% confidence intervals [CI]) for the risk of CVD in Q4 of the METS-IR and TyG index was 1.79 (1.25, 2.58) and 1.66 (1.28, 2.17), respectively (**Table 2**).

**Table 2:** Risk of cardiovascular diseases by baseline insulin resistance surrogates in Xinjiang’s rural population.

Variables	Model 1		Model 2		Model 3	
	HR (95% CI)	*P* value	HR (95% CI)	*P* value	HR (95% CI)	*P* value
METS-IR						
Continuous (per unit)	1.06 (1.05, 1.07)	<0.001	1.03 (1.02, 1.04)	<0.001	1.02 (1.01, 1.04)	0.005
Q1 (≤33.24)	Reference		Reference		Reference	
Q2 (33.25~)	2.16 (1.57, 2.98)	<0.001	1.58 (1.14, 2.18)	0.005	1.46 (1.05, 2.03)	0.026
Q3 (37.83~)	3.11 (2.29, 4.22)	<0.001	1.94 (1.42, 2.64)	<0.001	1.71 (1.23, 2.39)	0.002
Q4 (≥42.40)	4.77 (3.56, 6.40)	<0.001	2.24 (1.65, 3.04)	<0.001	1.79 (1.25, 2.58)	0.002
*P* for trend		<0.001		<0.001		0.004
TyG index						
Continuous (per unit)	2.12 (1.81, 2.48)	<0.001	1.70 (1.44, 2.01)	<0.001	1.64 (1.38, 1.94)	<0.001
Q1 (≤8.13)	Reference		Reference		Reference	
Q2 (8.14~)	1.60 (1.22, 2.11)	0.001	1.47 (1.12, 1.93)	0.006	1.49 (1.13, 1.96)	0.004
Q3 (8.50~)	1.77 (1.35, 2.31)	<0.001	1.42 (1.09, 1.86)	0.010	1.40 (1.06, 1.83)	0.016
Q4 (≥8.81)	2.47 (1.91, 3.18)	<0.001	1.79 (1.38, 2.31)	<0.001	1.66 (1.28, 2.17)	<0.001
*P* for trend		<0.001		<0.001		<0.001

Model 1: non-adjusted model.

Model 2: adjusted by baseline age and sex.

Model 3: adjusted by Model 2 and the baseline education level, exercise frequency, waist circumference, smoking, drinking, family history of cardiovascular disease, hypertension, high-density lipoprotein cholesterol, and low-density lipoprotein cholesterol.

Q1, Q2, Q3, and Q4 represent the four quartiles.

(HR, hazard ratio; CI, confidence interval; METS-IR, metabolic insulin resistance score; TyG index, triglyceride-glucose index).

**Figure 3:**
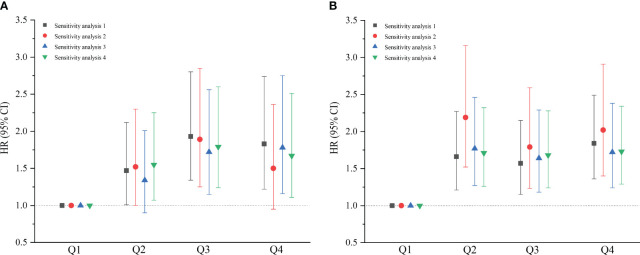
Sensitivity analyses of baseline metabolic insulin resistance score **(A)** and triglyceride-glucose index **(B)**. Sensitivity analysis 1 excluded the participants who experienced an outcome event within the first year of the follow-up (n=4602); Sensitivity analysis 2 excluded the population with hypertension (n=3609); Sensitivity analysis 3 excluded the population with dyslipidemia (n=3624); Sensitivity analysis 4 excluded the population with pre-diabetes and diabetes (n=4130). (*HR*, hazard ratio; *CI*, confidence interval; *Q1*, Quartile 1; *Q2*, Quartile 2; *Q3*, Quartile 3; *Q4*, Quartile 4).

The results of the sensitivity analyses were unchanged substantially after excluding those with an outcome event within the first year of follow-up, those with hypertension, those with dyslipidemia, and those with pre-diabetes and diabetes, in that order. This suggests that the results of this study are robust (**Figure 3**). The subgroup analysis indicated that the relationship between METS-IR and CVD was consistent with the main results, with an interaction elucidated between sex and METS-IR (*P*<0.05), males have a higher risk of CVD than females. The TyG index showed a positive association with the CVD risk in the subgroups of sex, age (<45 and ≥45 years), BMI (<28 and ≥28 kg/m^2^), and non-hypertensive population; however, in the hypertensive population, no connection between the TyG index and CVD was found to be statistically significant, as shown in an additional table file (**Supplementary Table 2**).

### 3.3 Characterization of the IR surrogates’ trajectories

**Figure 4:**
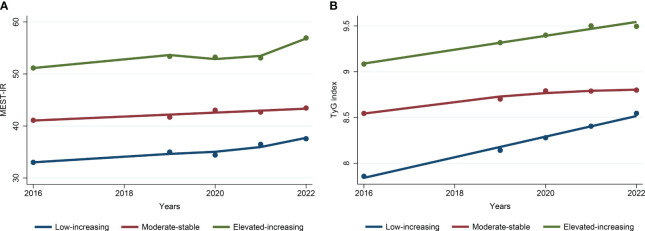
Trajectories of metabolic insulin resistance score **(A)** and triglyceride-glucose index **(B)** from 2016 to 2022. (*METS-IR*, metabolic insulin resistance score; *TyG* index, triglyceride-glucose index).

The trajectory analysis included 4343 participants, of which 1871 (43.1%) were male, and the average age was 39.09 ± 12.85 years, as shown in an additional table file (**Supplementary Table 3**). Based on the change trajectory patterns of METS-IR and TyG index from 2016 to 2022, this study revealed three distinct trajectories (**Figure 4**). According to the best-fitting trajectory model, the trajectory of METS-IR can be classified into the low-rising trajectory group (n=1985, 45.7%), moderate-stable trajectory group (n=2114, 48.7%), and elevated-increasing trajectory group (n=244, 5.6%). The trajectory of the TyG index can be classified into the low-rising trajectory group (n=556, 12.8%), moderate-stable trajectory group (n=3579, 82.4%), and elevated-increasing trajectory group (n=208, 4.8%). The average values of the observations at each time point for the different trajectory groups of the METS-IR and TyG index are shown in an additional table file (**Supplementary Table 4**).

### 3.4 Relationship between the IR surrogates’ trajectories and CVD

**Figure 5:**
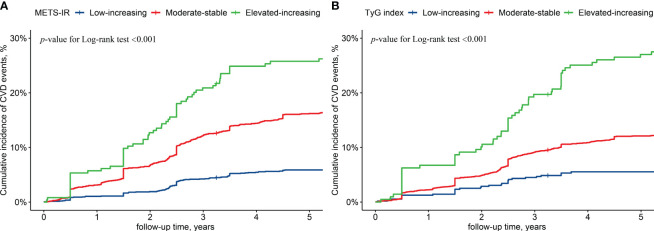
Cumulative cardiovascular diseases incidence by metabolic insulin resistance score **(A)** and triglyceride-glucose index **(B)** trajectories. (*METS-IR*, metabolic insulin resistance score; *TyG* index, triglyceride-glucose index; *CVD*, cardiovascular diseases).

The cumulative CVD incidence in the low-rising group of the METS-IR and TyG index was 5.9% and 5.6% respectively. The cumulative CVD incidence was 16.7% and 12.4% in the moderate-stable group and 25.8% and 27.4% in the elevated-increasing group of the METS-IR and TyG index, respectively. The elevated-increasing group had a greater cumulative incidence of CVD than the other trajectory groups according to the Kaplan–Meier curves (**Figure 5**). Using the low-rising group as a reference, the multifactorial analysis revealed that the HR (95% CI) for CVD risk in the moderate-stable group of the METS-IR and TyG index trajectories was 1.65 (1.30, 2.08) and 1.62 (1.12, 2.34), respectively. Moreover, the HR (95% CI) for CVD risk in the elevated-increasing group of the METS-IR and TyG index trajectories was 2.13 (1.48, 3.06) and 2.63 (1.68, 4.13), respectively (**Table 3**).

**Table 3:** Risk of incident cardiovascular diseases by the trajectory groups of the insulin resistance surrogates.

Variables	Case/Total	Model 1		Model 2		Model 3	
		HR (95% CI)	*P* value	HR (95% CI)	*P* value	HR (95% CI)	*P* value
METS-IR							
Low-increasing	118/1985	Reference		Reference		Reference	
Moderate-stable	352/2114	2.97 (2.41, 3.66)	<0.001	1.80 (1.45, 2.24)	<0.001	1.65 (1.30, 2.08)	<0.001
Elevated-increasing	63/244	4.95 (3.64, 6.72)	<0.001	2.55 (1.86, 3.49)	<0.001	2.13 (1.48, 3.06)	<0.001
TyG index							
Low-increasing	31/556	Reference		Reference		Reference	
Moderate-stable	445/3579	2.21 (1.54, 3.19)	<0.001	1.75 (1.22, 2.53)	0.003	1.62 (1.12, 2.34)	0.011
Elevated-increasing	57/208	5.23 (3.38, 8.10)	<0.001	2.85 (1.83, 4.45)	<0.001	2.63 (1.68, 4.13)	<0.001

Model 1: non-adjusted model.

Model 2: adjusted by baseline age and sex.

Model 3: adjusted by Model 2 and the baseline education level, exercise frequency, waist circumference, smoking, drinking, family history of cardiovascular disease, hypertension, high-density lipoprotein cholesterol, and low-density lipoprotein cholesterol.

(HR, hazard ratio; CI, confidence interval; METS-IR, metabolic insulin resistance score; TyG index, triglyceride-glucose index).

**Figure 6:**
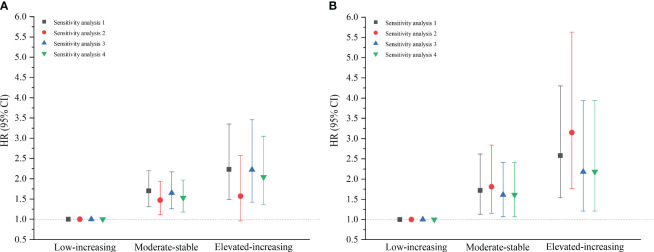
Sensitivity analyses of the metabolic insulin resistance score **(A)** and triglyceride-glucose index **(B)** trajectory groups. Sensitivity analysis 1 excluded the participants who experienced an outcome event within the first year of the follow-up (n=4242); Sensitivity analysis 2 excluded the population with hypertension (n=3334); Sensitivity analysis 3 excluded the population with dyslipidemia (n=3359); Sensitivity analysis 4 excluded the population with pre-diabetes and diabetes (n=3814). (*HR*, hazard ratio; *CI*, confidence interval).

Sensitivity analyses proved that on excluding individuals who experienced a CVD event within the first year of follow-up, those with hypertension, those with dyslipidemia, and those with pre-diabetes and diabetes, in that order, the relationship between the TyG index trajectory and CVD remained consistent with the primary results. The relationship between the METS-IR trajectory and CVD did not significantly change after excluding individuals experiencing a CVD event within the first year of follow-up and those with dyslipidemia. After excluding those with hypertension, no significant correlation was observed between the METS-IR trajectory and CVD incidence in the elevated-increasing group (**Figure 6**).

### 3.5 Predicting the CVD risk according to the baseline IR surrogates

The inclusion of the baseline METS-IR and TyG index to the Framingham model significantly increased its prediction potential. In the diverse sex populations, the addition of METS-IR and the TyG index enhanced the C-index. In the male population, the addition of the METS-IR and TyG index increased the NRI by 10.7% and 14.6%, respectively, and the IDI by 10.8% and 10.8%, respectively. In the female population, the addition of the METS-IR and TyG index increased the NRI by 18.1% and 22.7%, respectively, and the IDI by 0.9% and 1.3%, respectively (**Table 4**).

**Table 4:** Predictive value of baseline insulin resistance surrogates for cardiovascular diseases in Xinjiang’s rural population.

Model	C-index (95% CI)	*P* value	NRI (95% CI)	*P* value	IDI (95% CI))	*P* value
Male						
Framingham	0.782 (0.750, 0.814)	–	–	–	–	–
Framingham + METS-IR	0.811 (0.781, 0.841)	<0.001	0.107 (0.032, 0.183)	0.005	0.108 (0.087, 0.128)	<0.001
Framingham + TyG index	0.817 (0.788, 0.846)	<0.001	0.146 (0.072, 0.220)	<0.001	0.108 (0.089, 0.128)	<0.001
Female						
Framingham	0.729 (0.701, 0.757)	–	–	–	–	–
Framingham + METS-IR	0.740 (0.713, 0.767)	0.020	0.181 (0.129, 0.233)	<0.001	0.009 (0.006, 0.012)	<0.001
Framingham + TyG index	0.751 (0.724, 0.777)	<0.001	0.227 (0.170, 0.284)	<0.001	0.013 (0.009, 0.016)	<0.001

(NRI, net reclassification improvement; IDI, integrated discrimination improvement; CI, confidence interval; METS-IR, metabolic insulin resistance score; TyG index, triglyceride-glucose index).

## 4 Discussion

This study investigated the association of the baseline IR surrogates and their longitudinal trajectories with the risk of CVD in this regional population based on the prospective cohort study conducted in the rural districts of Xinjiang. The primary results of this study are described below. First, IR surrogates (METS-IR and TyG index) are predictors of the likelihood of CVD incidence, independent of the traditional risk factors. The risk of CVD increased with the ascending quartiles of the baseline IR surrogates. Second, the IR surrogate trajectories were classified into low-rising, moderate-stable, and elevated-increasing groups. For the METS-IR and TyG index, the potential risk of CVD was higher in the group with an elevated-increasing trajectory and lower in the group with a low-rising trajectory. Third, the addition of the baseline METS-IR and TyG index to the Framingham model increased the predictive value of CVD events.

A complex combination of genetic, metabolic, and environmental factors contributes to CVD. IR is a critical risk factor for CVD, and the formation of atherosclerotic plaque as well as anomalies of ventricular hypertrophy and diastolic function produced by IR can hasten the progression of CVD (19). The METS-IR and TyG index, recently introduced as alternatives to IR, have garnered interest in epidemiological studies for their simplicity of measurement and cost-effectiveness. The METS-IR is based on routine biochemical tests and BMI calculations, and it can be used to screen insulin sensitivity and assess the cardiometabolic risk for the early detection of healthy and high-risk populations (6). The TyG index, derived from fasting glucose and TG levels, is also being used as a simple alternative to IR because it has higher sensitivity and specificity than the IR gold standard method and HOMA-IR (5, 20–22). In several populations, the TyG index has been proven as a CVD predictor (23–28). Currently, research associating the IR surrogates with new-onset CVD at a single time point is commonly available; however, the studies are restricted in their ability to adequately reflect the impact of the longitudinal temporal changes in the IR surrogates on the development of CVD. The group-based trajectory model proposed by Nagin et al. (18) provides a thorough overview of IR development over time in the study population, identifies population subgroups with comparable patterns across time, and facilitates the identification of groups with a greater risk of CVD. Therefore, this study examined the relationship between baseline IR surrogates and CVD as well as the impact of the longitudinal trajectories of the IR surrogates on CVD.

### 4.1 IR surrogates were independent predictors of CVD

This study demonstrated that the baseline IR surrogates were independent risk factors for CVD and that higher IR surrogate values substantially enhanced the cumulative incidence of CVD. After multifactor adjustment, the HR (95% CI) for the risk of CVD in Q4 of the METS-IR and TyG index was 1.79 (1.25, 2.58) and 1.66 (1.28, 2.17), respectively, when compared with Q1. The subgroup analysis by sex revealed a statistically significant difference in the interaction between sex and METS-IR (*P*<0.05). The higher likelihood of CVD in males with high METS-IR than in their female counterparts was mostly related to the variations in the IR, lipid metabolism, and obesity levels between them. First, visceral fat is closely associated with IR. Males have a higher tendency to accumulate visceral fat, which is more harmful to human health than other areas of fat accumulation (29). Second, the decline of male gonadal activity increases with increased visceral fat and IR, which ultimately disturbs fat metabolism (30). Furthermore, no substantial changes were observed in the results of the sensitivity analyses after consecutively excluding those who had a CVD outcome event within the first year of follow-up, those with hypertension, those with dyslipidemia, and those with pre-diabetes and diabetes indicating that the findings of this study were relatively robust.

According to the literature on the Eastern Chinese population, a 1-unit elevation in the METS-IR can outcome in a 17% increase in the risk of CVD (31). Numerous studies examining the connection between IR surrogates and coronary artery calcification have reached the same conclusion that METS-IR has the strongest predictive value (32, 33). The overall HRs (95% CIs) for CVD risk in Q2, Q3, and Q4 of the TyG index were 1.05 (1.00, 1.10), 1.05 (1.00, 1.10), and 1.19 (1.14, 1.25), respectively, when compared with Q1, according to the UK Biobank cohort study with a sample size of over 4,00,000 (34). The TyG index had a substantial correlation with the onset of CVD, which was an independent predictor of CVD risk in the Caucasian population, according to the findings of the VMCUN cohort (35). Although the average age of the participants in our study was lesser than that in the aforementioned studies, both METS-IR and TyG index were equally established as independent risk factors for CVD. The METS-IR and TyG index can be regarded as essential reference indicators to indicate the CVD risk in the rural areas of Xinjiang, but we should carefully implement them within clinical practice, and clinical factors, such as blood glucose and blood lipid indicators, should also be considered in the use process. Furthermore, CVD is a disease with a multitude of pathogenic factors, which should be considered in conjunction with factors such as obesity, blood glucose, and blood lipid when stratifying the management of at-risk populations.

### 4.2 Long-term trajectories of elevated IR surrogates were associated with a high risk of CVD

The trajectory characteristics for each subgroup of the IR surrogates in this population were determined for the first time in this study, and the trajectories of METS-IR and TyG index were classified into the low-rising, moderate-stable, and elevated-increasing groups. These three trajectory groups of the METS-IR had a cumulative CVD incidence of 5.9%, 16.7%, and 25.8%, respectively, while those of the TyG index had a cumulative CVD incidence of 5.6%, 12.4%, and 27.4%, respectively. According to the Kaplan–Meier curves, the elevated-increasing group had a higher cumulative incidence of CVD than the other trajectory groups. Based on the multifactorial Cox model results, the CVD risk was 1.65 and 2.13 times greater in the moderate-stable and elevated-increasing groups than in the low-rising group of the METS-IR, respectively. The risk of CVD was 1.62 and 2.63 times greater in the moderate-stable and elevated-increasing groups than in the low-rising group of the TyG index, respectively. The Hanzhong adolescent hypertension cohort study revealed three TyG index trajectories and concluded that long-term trajectories of elevated TyG index were independently related to increased arterial stiffness (36). The same conclusion was confirmed that continuously increasing TyG index trajectories are associated with a noticeably higher risk of CVD in populations with normal weight, hypertension, and diabetes (37–39). For the early detection of CVD in the rural population of Xinjiang, it is essential to complete relevant biochemical indicator measurements and conduct long-term follow-ups. Individuals with elevated immediate levels of METS-IR and TyG index, along with those with elevated longitudinal trajectory levels should be actively followed.

### 4.3 IR surrogates had predictive value for the risk of CVD

Based on the Framingham risk score model, the predictive power of the baseline IR surrogates for CVD risk was evaluated in this study. After including the baseline METS-IR and TyG index in the Framingham model, significant improvements were observed in the new models of C-index, NRI, and IDI (*P*<0.05). The C-index increased by 0.009 in the investigation of METS-IR and coronary artery calcification in asymptomatic adults when the METS-IR was introduced to the prior model (*P*<0.001) (32). According to research on the population without diabetes in the East China region, the TyG index considerably enhanced the predictive value of CVD in the new model, when it was included in one that previously comprised only the traditional risk variables (40). Wang et al. (41) proved that the addition of the TyG index to a model with conventional risk factors could enhance its predictive ability of the risk of CVD. The results of this study corroborate the findings of the aforementioned studies.

## 5 Benefits and limitations

The primary strengths of this study are based on the aspects described below. The prospective cohort study approach showed high strength for causal justifications. It was representative of the general situation of the population of rural Xinjiang. It considered the implications of the dynamic changes in the METS-IR and TyG index on CVD during follow-up and revealed the regularity of the development of the METS-IR and TyG index in this study population. Multiple confounding factor adjustments were made regarding the association of the baseline METS-IR and TyG index along with their longitudinal trajectories with new-onset CVD. Moreover, sensitivity analyses were conducted to validate the study results.

This study has some limitations. First, with the characteristics of a long onset, complex course, lifelong nature, and uncertainty, CVD should be followed up over an extended period to validate the study findings. Second, there were some potential residual confounders, and the effects of economic income and nutritional intake on the study were not considered. Third, since fasting insulin levels in the study population were not evaluated, the effects of the IR surrogates on CVD risk could not be compared to those of HOMA-IR. Finally, since this study was limited to the population of the rural areas of Xinjiang, the generalization of its findings to other groups should be conducted cautiously.

## 6 Conclusion

Elevated baseline METS-IR and TyG index values were independent risk factors for new-onset CVD in the Xinjiang rural population. Furthermore, METS-IR and TyG index trajectory patterns were intimately linked to an increased risk of CVD. Glucose metabolism status can be assessed by utilizing METS-IR and TyG index in large-scale epidemiological surveys for identifying individuals at a high risk of CVD, considering its user-friendly assessment, simplicity of calculation, and low cost.

## Data availability statement

The raw data supporting the conclusions of this article will be made available by the authors, without undue reservation.

## Ethics statement

The studies involving humans were approved by The Institutional Ethics Review Board of the First Affiliated Hospital of Shihezi University Medical College. The studies were conducted in accordance with the local legislation and institutional requirements. The participants provided their written informed consent to participate in this study.

## Author contributions

SW: Investigation, Conceptualization, Data curation, Methodology, Software, Visualization, Writing – original draft. XZ: Conceptualization, Data curation, Investigation, Writing – original draft. MK: Conceptualization, Data curation, Investigation, Writing – review & editing. HG: Data curation, Investigation, Project administration, Resources, Writing – review & editing. JH: Investigation, Methodology, Resources, Writing – review & editing. ReM: Conceptualization, Data curation, Investigation, Writing – review & editing. XW: Conceptualization, Data curation, Investigation, Writing – review & editing. RuM: Investigation, Supervision, Writing – review & editing. SG: Funding acquisition, Investigation, Project administration, Resources, Supervision, Writing – review & editing.
