# Supplementary material for: Impact of the baseline insulin resistance surrogates and their longitudinal trajectories on cardiovascular disease (coronary heart disease and stroke): a prospective cohort study in rural China
Source: Front Endocrinol (Lausanne). 2023 Dec 22;14:1259062. doi: 10.3389/fendo.2023.1259062 (PMC10767254; doi:10.3389/fendo.2023.1259062)
Supplement: Supplementary file 1 [file Table_1.pdf]

Supplementary Material

Impact of the baseline insulin resistance surrogates and their longitudinal trajectories on cardiovascular disease outcomes: A prospective cohort study in rural China

Shulin Wang,[†] Xianghui Zhang,[†] Mulatibieke Keerman, Heng Guo,Jia He, Remina Maimaitijiang, Xinping Wang, Rulin Ma,[*] Shuxia Guo[*]

†: These authors contributed equally to this work.

*** Correspondence:**

Rulin Ma: marulin@shzu.edu.cn, Shuxia Guo: gsxshzu@sina.com

1.Supplementary Tables

Supplementary Table 1 The Bayesian information criterion of the different models of the insulin resistance surrogates

Variables	Model	Parameters of trajectory shape	BIC	Probability of groups (%)					Average posterior probability
				Group 1	Group 2	Group 3	Group 4	Group 5	
METS-IR	2	33	-44188.80	73.97	26.03				0.95/0.88
	3	333	-43471.58	45.16	48.92	5.92			0.88/0.89/0.92
	4	3333	-43256.92	26.19	51.04	19.39	3.37		0.85/0.83/0.83/0.93
	5	33333	-43135.31	27.15	50.97	7.53	11.34	3.01	0.87/0.84/0.75/0.76/0.93
	**3**	**313**	**-43464.59**	**45.22**	**48.87**	**5.91**			**0.88/0.89/0.92**
TyG index	2	33	-9278.94	31.34	68.66				0.79/0.86
	3	333	-9104.11	15.86	78.06	6.08			0.79/0.90/0.82
	4	3333	-9080.98	5.38	65.77	23.88	4.96		0.75/0.83/0.65/0.82
	5	33333	-9077.23	7.51	4.54	18.98	63.78	5.19	0.69/0.59/0.57/0.81/0.82
	**3**	**121**	**-9093.96**	**15.75**	**78.23**	**6.02**			**0.79/0.91/0.81**

(*BIC*, Bayesian information criterion)

Supplementary Table 2 Subgroup analyses of baseline insulin resistance surrogates and cardiovascular diseases risk in Xinjiang's rural population

**Variables**	**Subgroups**	**Q1**	**Q2**	**Q3**	**Q4**	***P* for interaction**
METS-IR	Sex					0.002
	Male	Reference	1.21 (0.67, 2.19)	2.04 (1.15, 3.64)	2.46 (1.33, 4.53)	
	Female	Reference	1.54 (1.03, 2.29)	1.41 (0.93, 2.14)	1.52 (0.96, 2.39)	
	Age (years)					0.680
	≥45	Reference	1.74 (1.06, 2.85)	1.72 (1.04, 2.83)	1.34 (0.76, 2.36)	
	<45	Reference	1.63 (1.16, 2.30)	1.76 (1.22, 2.52)	2.41 (1.64, 3.54)	
	BMI (kg/m^2)					0.140
	≥28	Reference	1.23 (0.81, 1.89)	1.75 (1.15, 2.64)	1.66 (1.05, 2.63)	
	<28	Reference	1.44 (0.91, 2.29)	1.83 (1.17, 2.88)	2.52 (1.57, 4.04)	
	Hypertension					0.350
	Yes	Reference	1.51 (0.95, 2.41)	2.03 (1.27, 3.25)	2.68 (1.59, 4.53)	
	No	Reference	1.52 (1.00, 2.30)	1.89 (1.25, 2.85)	1.50 (0.95, 2.36)	
TyG index	Sex					0.250
	Male	Reference	1.67 (1.06, 2.63)	1.23 (0.76, 1.98)	1.86 (1.20, 2.87)	
	Female	Reference	1.85 (1.29, 2.65)	1.88 (1.32, 2.68)	1.81 (1.27, 2.57)	
	Age (years)					0.200
	≥45	Reference	1.69 (1.23, 2.32)	1.32 (0.95, 1.84)	1.90 (1.38, 2.60)	
	<45	Reference	1.74 (1.15, 2.64)	1.54 (1.00, 2.38)	1.51 (0.98, 2.31)	
	BMI (kg/m^2)					0.870
	≥28	Reference	1.40 (0.97, 2.03)	1.34 (0.93, 1.93)	1.61 (1.12, 2.32)	
	<28	Reference	2.14 (1.42, 3.21)	1.97 (1.31, 2.97)	2.21 (1.48, 3.29)	
	Hypertension					0.070
	Yes	Reference	1.18 (0.79, 1.76)	1.16 (0.78, 1.72)	1.47 (0.99, 2.17)	
	No	Reference	2.19 (1.52, 3.16)	1.79 (1.23, 2.59)	2.02 (1.40, 2.91)	

Models adjusted by baseline age, sex, education level, exercise frequency, waist circumference, smoking, drinking, family history of cardiovascular diseases, high-density lipoprotein cholesterol, and low-density lipoprotein cholesterol.
Q1, Q2, Q3, and Q4 represent the four quartiles, respectively.
(*METS-IR*, metabolic insulin resistance score; *TyG* index, triglyceride-glucose index)

Supplementary Table 3 Baseline characteristics of trajectories in Xinjiang's rural population according to insulin resistance surrogates tajectory groups

**Variables**	**Total (n=4343)**	**METS-IR index trajectory groups**					**TyG index trajectory groups**				
		**Low-increasing （n=1985）**	**Moderate-stable (n=2114)**	**Elevated-increasing (n=244)**	**F/χ^2 value**	***P* value**	**Low-increasing （n=556）**	**Moderate-stable (n=3579)**	**Elevated-increasing (n=208)**	**F/χ^2 value**	***P* value**
Male (%)	1871 (43.1)	909 (45.8)	888 (42.0)	74 (30.3)	23.137	<0.001	152 (27.3)	1599 (44.7)	120 (57.7)	78.024	<0.001
Age (years)	39.09±12.85	33.92±11.64	43.19±12.23	45.73±11.52	349.291	<0.001	34.90±10.93	39.27±12.86	47.19±13.11	73.584	<0.001
Education level					92.355	<0.001				15.922	0.003
Illiterate/semi-illiterate (%)	1708 (39.3)	641 (32.3)	935 (44.2)	132 (54.1)			201 (36.2)	1402 (39.2)	105 (50.5)		
Junior high school and below (%)	2317 (53.4)	1191 (60.0)	1019 (48.2)	107 (43.9)			308 (55.3)	1913 (53.4)	96 (46.2)		
High School and above (%)	318 (7.3)	153 (7.7)	160 (7.6)	5 (2.0)			47 (8.5)	264 (7.4)	7 (3.3)		
Exercise frequency					11.002	0.027				17.122	0.002
Regular exercise (%)	1435 (33.0)	622 (31.3)	736 (34.8)	77 (31.6)			204 (36.7)	1166 (32.5)	65 (31.3)		
Occasional exercise (%)	342 (7.9)	178 (9.0)	150 (7.1)	14 (5.7)			62 (11.1)	267 (7.5)	13 (6.2)		
Almost no exercise (%)	2566 (59.1)	1185 (59.7)	1228 (58.1)	153 (62.7)			290 (52.2)	2146 (60.0)	130 (62.5)		
Systolic blood pressure (mmHg)	127.76±20.09	121.57±16.65	132.23±21.03	139.31±21.99	204.375	<0.001	122.25±18.05	128.10±20.00	136.45±22.72	41.635	<0.001
Diastolic blood pressure (mmHg)	75.64±12.38	72.34±10.49	77.92±12.89	82.75±14.59	157.007	<0.001	73.63±11.03	75.77±12.48	78.76±13.41	14.223	<0.001
FPG (mmol/L)	4.71±1.03	4.60±0.85	4.80±1.11	4.93±1.35	25.540	<0.001	4.23±0.59	4.71±0.76	6.03±2.95	262.712	<0.001
TG (mmol/L)	1.41±0.63	1.23±0.58	1.54±0.62	1.72±0.67	175.234	<0.001	0.72±0.25	1.46±0.54	2.32±0.87	783.868	<0.001
TC (mmol/L)	4.64±1.04	4.43±0.99	4.81±1.05	4.75±1.02	72.223	<0.001	3.95±0.76	4.69±1.01	5.52±1.19	222.662	<0.001
HDL-C (mmol/L)	1.42±0.38	1.51±0.42	1.35±0.33	1.23±0.28	124.739	<0.001	1.38±0.38	1.42±0.38	1.41±0.37	3.514	0.030
LDL-C (mmol/L)	2.59±0.89	2.41±0.87	2.72±0.87	2.93±0.92	80.126	<0.001	2.52±0.87	2.58±0.89	2.98±0.88	22.767	<0.001
BMI (kg/m^2)	26.59±4.12	23.52±2.47	28.58±2.79	34.24±3.94	2784.465	<0.001	24.80±4.03	26.78±4.06	28.08±3.98	71.877	<0.001
WC (cm)	91.16±11.24	85.16±9.59	95.29±9.41	104.26±11.08	812.161	<0.001	85.37±11.91	91.83±10.85	95.17±11.02	97.529	<0.001
Smoking (%)	619 (14.3)	310 (15.6)	286 (13.5)	23 (9.4)	8.581	0.014	52 (9.4)	515 (14.4)	52 (25.0)	30.637	<0.001
Drinking (%)	164 (3.8)	79 (4.0)	81 (3.8)	4 (1.6)	3.311	0.191	4 (0.7)	146 (4.1)	14 (6.7)	20.200	<0.001
Hypertension (%)	1009 (23.2)	247 (12.4)	659 (31.2)	103 (42.2)	253.581	<0.001	83 (14.9)	847 (23.7)	79 (38.0)	47.243	<0.001
Family history of CVD (%)	541 (12.5)	252 (12.7)	253 (12.0)	36 (14.8)	1.748	0.417	85 (15.3)	430 (12.0)	26 (12.5)	4.728	0.094

(*CVD*, cardiovascular diseases; *FPG*, fasting plasma glucose; *TG*, triglycerides; *TC*, total cholesterol; *LDL-C*, low-density lipoprotein cholesterol; *HDL-C*, high-density lipoprotein cholesterol; *BMI*, body mass index, *WC*, waist circumference)

Supplementary Table 4 Each time point observation of the insulin resistance surrogates' trajectory groups in Xinjian's rural population

Time points	METS-IR index trajectory groups			TyG index trajectory groups		
	**Low-increasing**	**Moderate-stable**	**Elevated-increasing**	**Low-increasing**	**Moderate-stable**	**Elevated-increasing**
T1	32.73±3.98	41.47±4.56	51.46±6.44	7.70±0.39	8.54±0.40	9.18±0.49
T2	34.83±4.31	41.93±4.26	53.80±6.57	8.03±0.47	8.71±0.34	9.44±0.24
T3	34.20±4.25	43.34±4.80	53.48±5.29	8.20±0.49	8.79±0.44	9.51±0.43
T4	36.29±4.13	42.91±4.81	53.64±7.84	8.35±0.43	8.79±0.41	9.62±0.52
T5	37.38±4.23	43.67±5.21	57.40±7.43	8.50±0.39	8.80±0.38	9.61±0.50

(*METS-IR*, metabolic insulin resistance score; *TyG* index, triglyceride-glucose index)
